# A Modification of the Clinical Thermometer

**Published:** 1868-10

**Authors:** S. H. Frazer


### A MODIFICATION OF THE CLINICAL THERMOMETER.

Presented to the Medical Society of the Hospitals of Paris, by M. HERARD.

At this time, when thermometrical researches are in vogue, I thought that the Society would receive with interest the modifications of the thermometer, used as a means of ascertaining the temperature of the body in disease. The instrument is constructed by MM. Alvergniot frères, under the instructions of M. Niederkom, a pupil attached to my service.

The instrument which I present contains mercury, with calibre equally and carefully prepared, with a fractional scale, and graduated on glass, by degrees and tenths of degrees. It is "*maximum in all directions, with a permanent bubble of air,*" not requiring any manipulations to prepare a maximum, nor any calculation for the reading. The constructor has broken the column of mercury, by the introduction of a very fine bubble of air, above which a fraction of the column remains, serving as an index, and occupying about ten divisions. The instrument is about the size of an ordinary lead pencil; it is about six inches in length, of which three are occupied by the reservoir. The reservoir alone is blown; the body is plain, except the capillary tube, which passes longitudinally through its centre, giving to the instrument all the solidity the material will admit of.

The inferior half of the instrument is not graduated. The superior half is graduated between 34° and 42° (Cent.). This scale suffices for ordinary cases, but it can be made, on the same system, to meet any requirements.

When the index is below the temperature we think we ought to obtain, there is nothing to do but put the instrument in position; if, on the contrary, a preceding observation has shown the index, we must preliminarily arrange the index below the expected temperature, which is accomplished by slight shakings of the instrument.

After two or three minutes, when the equilibrium of temperature has become established, withdraw it, and the reading is very easily accomplished. "*The index remaining fixed by capillary adhesion at the highest point of the course through which it has just passed.*" The instrument has been so graduated that in order to appreciate exactly the temperature, it is only necessary to read the division just above the level of the index. The scale having only been constructed for ordinary diseases, it is not very long. The divisions have consequently been given in tenths, so as not to make the size inconvenient. By employing mercury, the exact calibre of the tube, and graduation on glass, the skilful operator has been enabled to produce very precise instruments, the sensibility of which is due to the form, to the small capacity of the reservoir, and above all, to the extreme capillarity of the tube. For more than two months M. Niederkom has used it daily under my personal observation, and it performs with perfect regularity. In addition to its above-mentioned qualities, this advantage can be claimed for it over the ordinary instrument, viz., "*that the reading is no longer made on the patient, and can be deferred.*" Thus it is always exact and convenient, despite its extreme capillarity, because we can always seek the most favorable spot for the reading where the light is strongest.

With the instrument in use to-day, we meet with inevitable causes for cooling the instrument; for we must either leave a portion of the body of the instrument exposed to the air in maintaining an opening between the skin and clothing during the entire observation, or place the reservoir in the axilla, the entire body applied along the side by means of the arm, and carefully cover the patient. This procedure gives a just equilibrium, but at the moment when we wish to ascertain the temperature, we uncover the patient in order to bring the body in sight, and then we expose ourselves to the same causes of error as before. These objections disappear in employing this method of the maximum thermometer; for once the equilibrium is registered, it can not change, grace to the index. Thus disappear also, in great part at least, the reasons given generally for preferring the rectal temperature in hospital practice.

To place the instrument in position and withdraw it is a manœuvre so simple, that the patient himself or any one of his family can perform it without any chance of error. Then it can be applied without any trouble on the most modest lady without shocking her delicacy.—*S. H. Frazer.*—*L'Union Medicale*, 1868.

